# Impact of the COVID-19 pandemic on alcohol or drug use symptoms and service need among youth: a cross-sectional sample from British Columbia, Canada

**DOI:** 10.1186/s13011-022-00508-9

**Published:** 2022-12-22

**Authors:** Kirsten Marchand, Guiping Liu, Emilie Mallia, Nikki Ow, Krista Glowacki, Katherine G. Hastings, Steve Mathias, Jason M. Sutherland, Skye Barbic

**Affiliations:** 1 Foundry, 915-1045 Howe Street, Vancouver, BC V6Z 2A9 Canada; 2 Faculty of Medicine, University of British Columbia, 317-2194 Health Sciences Mall, Vancouver, BC V6T 1Z3 Canada; 3 Department of Occupational Science and Occupational Therapy, University of British Columbia, 317-2194 Health Sciences Mall, Vancouver, BC V6T 1Z3 Canada; 4 Centre for Health Evaluation & Outcome Sciences, 588-1081 Burrard Street, Vancouver, BC V6Z 1Y6 Canada; 5 Centre for Health Services and Policy Research, 201- 2206 East Mall, Vancouver, BC V6T 1Z3 Canada; 6 Department of Psychiatry, University of British Columbia, 2255 Wesbrook Mall, Vancouver, BC V6T 2A1 Canada; 7 School of Population and Public Health, University of British Columbia, 2206 East Mall, Vancouver, BC V6T 1Z3 Canada

**Keywords:** Youth, Adolescents, Young adults, COVID-19 pandemic, Substance-related disorders, Early intervention

## Abstract

**Background:**

Concerns about youth alcohol and drug use have risen since the declaration of the global COVID-19 pandemic due to the pandemic’s impact on known risk and protective factors for substance use. However, the pandemic’s immediate and long-term impact on youths’ substance use patterns has been less clear. Thus, this study sought to determine how the COVID-19 pandemic impacted alcohol or drug use and its risk and protective factors among youth accessing integrated youth services.

**Methods:**

We conducted a repeated cross-sectional study of patient-reported outcomes data collected between May 2018 and February 2022 among youth (*n* = 6022) ages 10–24 accessing a provincial network of integrated youth services in Canada. The main exposure of interest was the COVID-19 pandemic (March 2020 – February 2022) compared with a pre-pandemic period (May 2018 – February 2020). As measured by the Global Appraisal of Individual Needs – Short Screener, outcomes included the average number of past month alcohol or drug use symptoms and past month likelihood of service need for alcohol/drug use (moderate/high vs. low need). Interrupted time series (ITS) examined change in average monthly alcohol/drug use symptoms between the pre- and pandemic periods. Stratified multivariable logistic regression investigated how the pandemic modified the effects of established risk/protective factors on likelihood of alcohol/drug use service need.

**Results:**

Fifty-percent of youth met the criteria for moderate/high likelihood of alcohol/drug use service need, with the odds being 2.39 times (95% confidence interval = 2.04, 2.80) greater during the pandemic compared to the pre-pandemic period. Results from the ITS indicated significant immediate effects of the pandemic on monthly substance use symptoms (*p* = 0.01). Significant risk/protective factors for service need included exposure to violence, engagement in meaningful activities, and self-rated physical and mental health; and the direction of their effects remained consistent across pandemic and pre-pandemic periods.

**Conclusions:**

This study demonstrated that the COVID-19 pandemic corresponded with increased alcohol or drug use among youth accessing integrated services. This signals an urgent need for increased clinical capacity in existing youth services and policies that can respond to risk/protective factors for substance use earlier.

**Supplementary Information:**

The online version contains supplementary material available at 10.1186/s13011-022-00508-9.

## Background

Substance use among youth aged 10–24 years is a global health priority [1]. Substance use typically emerges and peaks during this developmental period [1, 2] and can have lifelong impacts on a young person, including social and economic harms, poor physical and mental health, and a higher risk for developing substance use disorders [3]. Typically, efforts to reduce youth substance use focus on addressing widely known risk and protective factors through prevention and early intervention [4]. These factors can predict an increased likelihood of substance use (risk factors) or a lower likelihood of substance use by promoting or protecting exposure to the risk factors (protective factors) [5]. Risk and protective factors can be fixed (e.g., gender, race/ethnicity), contextual (e.g., social norms, substance availability), or individual and interpersonal (e.g., mental health, employment, family relations) [5, 6].

Given the importance of these risk and protective factors, concerns about youth substance use have risen since the declaration of the global COVID-19 pandemic in March 2020 [7–10]. The pandemic’s public health measures (e.g., stay-at-home orders, physical distancing) have dramatically impacted the risk and protective factors. For instance, many youth have experienced substantial increases in household stress and family conflict and reduced opportunities for employment and academic, recreational, and social connections [11, 12]. The pandemic has also been associated with an increased prevalence of mental health disorders and symptoms among youth (e.g., distress, depression, anxiety) [7, 12, 13], which have been linked to changes in risk and protective factors [12] and can increase risk for substance use [5]. However, the immediate impacts of the pandemic on youth substance use, as speculated [8, 10], has been less clear [9].

A recent systematic review of 49 studies examining the prevalence of alcohol, cannabis, tobacco, e-cigarettes/vaping, and drug use among youth during the COVID-19 pandemic found mixed results [9]. For example, among the 32 studies investigating alcohol use, five studies reported an increase, 12 reported a decrease, four reported no change and 11 reported neither an increase or decrease [9]. A similar mix of results was reported for the other substance types. While the review generally concluded that substance use declined among youth during the pandemic, differences between the original study’s designs and their findings make it difficult to draw such conclusions. In particular, the original studies measured different types of substances (tobacco, alcohol, cannabis, other drugs), a combination of substances, or did not specify substance type. Substance use outcomes were also measured for observation periods (i.e., early in the pandemic, during specific lockdown measures) that preclude understanding of temporal trends of substance use throughout the pandemic. Lastly, several studies measured substance use outcomes for reference periods (i.e., past year use, past 3-month use) that may introduce measurement error due to temporal ambiguity regarding the pandemic (exposure) and substance use (outcome).

Crucially, few existing studies systematically integrated known risk and protective factors into their designs [14–16], despite their established effects on youth substance use and the pandemic’s impact on these factors. One early pandemic study found that adolescents’ alcohol and cannabis use increased, with peer-reputation concerns being significant predictors of their social contexts for substance use (i.e., alone, with friends via technology) [16]. Additionally, a study by Romm et al., [15] found adverse childhood events, depression scores, and job losses during the pandemic predicted increases in substance use among young adults, with resilience being a significant moderator of their effects. Such studies are critical to understanding the pandemic’s full effects on youth substance use, identifying youth who may need substance use interventions, and informing ongoing intervention efforts.

Accordingly, the present study was designed to address some of the limitations of those prior studies by analyzing monthly changes to alcohol or drug use over a 4-year observation period and by systematically incorporating risk and protective factors to the study design. The primary objective of this study was to determine how the COVID-19 pandemic impacted past month alcohol or drug use and the risk and protective factors for alcohol or drug use among youth seeking integrated health and social services. We hypothesized that the pandemic increased the prevalence of alcohol or drug use in this sample, negatively affected risk and protective factors and modified their effect on alcohol or drug use. The results of this study provide more comprehensive evidence on the pandemic’s impact on alcohol or drug use among youth seeking healthcare services. These findings are timely as countries emerge from the pandemic and need to plan, design, and implement interventions for youth substance use.

## Methods

### Design and setting

This study is based on a repeated cross-sectional sample of youth (ages 10–24) accessing Foundry, an integrated youth service (IYS) network in the province of British Columbia (BC), Canada [17]. Briefly, Foundry is a network of 13 community-based IYS centres that have been expanding throughout BC since 2018. Foundry centres integrate five core service streams, including physical and sexual health, mental health, substance use, and social and peer support services [17]. These services are delivered by interdisciplinary healthcare professionals (physicians, nurses, counselors, and peer support specialists).

### Sample and data set

The analytic sample for this study included youth (*n* = 6022) who sought/received IYS and voluntarily completed a comprehensive set of patient reported outcomes (PROs) during the study period, May 1, 2018 to February 23, 2022. PROs were collected from youth at one time point, at the time of their first service request/visit, to inform IYS design and delivery and for research and evaluation. The PROs include clinical screening scales and questionnaires regarding youths’ demographic characteristics, social determinants of health, and physical health, mental health and substance use outcomes. From this data set, monthly cross-sectional data on key measures of interest (further detailed below) were generated. Data were accessed upon ethics approval from the Providence Health Care/University of British Columbia Research Ethics board (H22–00522).

### Exposures and outcomes measures

The main exposure was the COVID-19 pandemic (March 1, 2020 – February 23, 2022) vs. the pre-pandemic (May 1, 2018 – February 28, 2020). To measure the impact of the pandemic on risk/protective factors for substance use, additional exposures were selected based on established frameworks and empirical literature [6, 18], but were limited to availability of measured PROs. These risk/protective factors included social and environmental factors (education/employment, financial stress, current housing situation, safety in current housing, family support, engagement in meaningful activities, exposure to violence), and health-related factors (self-rated physical health, self-rated mental health).

Alcohol or drug use outcomes were measured using the past month substance use subscale of the Global Appraisal of Individual Needs – Short Screener (GAIN-SS) [19]. Aligning with common symptoms used to diagnose substance use disorders, the GAIN-SS substance use subscale includes five questions regarding the last time youth used alcohol or drugs weekly and were impacted by their alcohol or drug use (e.g., spent a lot of time using alcohol/drugs, kept using alcohol/drugs despite it causing social problems, experienced withdrawal symptoms, etc). Scores range from 0 to 5 with a higher score indicating a higher number of past month symptoms [19]. From this scale, two outcome variables were selected. First, we examined the average number of past month alcohol/drug use symptoms per youth (range 0–5), as a measure of symptomatolgy. Second, we analyzed the likelihood of alcohol/drug use service need as a diagnostic measure. Youth were categorized into two clinical groups according to scale norms, reflecting: (1) youth with a low likelihood of service need (count of 0 alcohol/drug use symptoms in the past month); and (2) youth with a moderate/high likelihood of service need (count of 1–5 alcohol/drug use symptoms in the past month) [19].

### Statistical analysis

Descriptive (frequencies) and bivariate (chi-square) statistics were used to describe and compare the demographic and substance use characteristics of youth completing the PROs measures during the pre- and post-pandemic periods. Interrupted time series (ITS) regression was used to analyze whether the pandemic was associated with a change in the average monthly substance use symptoms. This model generates three time-based estimates: (1) the monthly trend/slope in average substance use symptoms before the pandemic; (2) the level change in average substance use symptoms in the month immediately after the pandemic, accounting for the pre-pandemic trend; and (3) the change in trend/slope after the pandemic [20, 21]. In our ITS, the pre-pandemic period included 22 months of data (May 2018 – February 2020) and the pandemic period (the event of interest) included 24 months (March 2020 – February 2022). This design provided an approximately equal number of months of data before and after the pandemic and an adequate number of data points to detect a change in average monthly substance use symptoms and allow adjustment for seasonality [21].

Logistic regression then analyzed the association between risk/protective factors and the likelihood of alcohol/drug use service need, adjusting for age, gender, and race. All risk/protective factors were initially considered in univariable logistic regression models (Additional File 1, Table 1). For the final multivariable model, two variables (financial stress and current housing situation) were excluded due to collinearity, leaving a total of 11 variables. This model was then stratified by pre-pandemic (*n* = 5066) and pandemic (*n* = 956) samples to explore how the pandemic modified the effects of each risk/protective factor on alcohol/drug use service need. Since many of the risk/protective factors in the final multivariable model were variables with > 2 categories, contrasts tests were performed as a supplementary analysis to estimate their overall association with moderate/high likelihood of alcohol/drug use need (Additional File 1, Table 2). While overall item non-response was low for the risk/protective factors, it was not consistent across the pandemic vs. pre-pandemic periods and thus, data may be missing at random. To reduce further loss of statistical power for the stratified analyses, we used the missing-indicator method [22], which yielded similar results as a complete case sensitivity analysis (Additional file 1, Table 3). All analyses were performed using two-tailed tests with an alpha level of 0.05 and conducted in SAS, version 9.4.

## Results

### Youths’ demographic characteristics, substance use patterns, and risk/protective factors during pre- and pandemic-periods

Tables 1 and 2 summarize results from the descriptive analysis of youths’ demographic and substance use characteristics and the select risk and protective factors, with comparisons between the pre- and pandemic periods. As shown in Table 1, a high proportion of youth overall were in the 20–24 age category (49%) and identified as women (57%) and White (64%). Regarding youths’ substance use characteristics, significant differences between the pandemic vs. pre-pandemic periods were observed. Of note, a higher proportion of youth reported ever using illicit drugs (50% vs. 35%) and regular use of cannabis (40% vs. 26%) in the pandemic compared to pre-pandemic period. Additionally, a higher proportion of youth met criteria for high past year (49% vs. 32%) and past month (27% vs. 19%) likelihood of substance use service need in the pandemic vs. pre-pandemic period.

**Table 1:** Characteristics of youth accessing integrated youth services during the pre-pandemic and pandemic periods

Characteristic	Overall	Pre-pandemic	Pandemic	Chi-square	DF	*p-*value
	n = 6022 No. (%)	n = 5066 No. (%)	n = 956 No. (%)			
Demographic Characteristics						
Age group:				9.68	3	0.021
10–14	711 (12)	625 (12)	86 (9)			
15–19	2281 (38)	1902 (37)	379 (40)			
20–24	2972 (49)	2493 (49)	479 (50)			
Missing	58 (1)	46 (1)	12 (1)			
Gender identity:				8.42	2	0.015
Man	2033 (34)	1699 (33)	334 (35)			
Woman	3444 (57)	2929 (58)	515 (54)			
Gender diverse/other ^a^	545 (9)	438 (9)	107 (11)			
Race: ^b^				19.86	2	< 0.001
White	3870 (64)	3316 (65)	554 (58)			
Non-white	2031 (34)	1653 (33)	378 (40)			
Missing	121 (2)	97 (2)	24 (2)			
Lifetime or past year substance use patterns						
Lifetime prescription drug use: ^c^				13.93	2	0.001
Yes	1740 (29)	1429 (28)	311 (32)			
No	3697 (61)	3119 (62)	578 (61)			
Missing	585 (10)	518 (10)	67 (7)			
Lifetime illicit drug use: ^d^				75.84	2	< 0.001
Yes	2250 (37)	1774 (35)	476 (50)			
No	3187 (53)	2774 (55)	413 (43)			
Missing	585 (10)	518 (10)	67 (7)			
Lifetime injection drug use:				9.58	2	0.008
Yes	110 (2)	93 (2)	17 (2)			
No	5555 (92)	4652 (92)	903 (94)			
Missing	357 (6)	321 (6)	36 (4)			
GAIN-SS past year likelihood of substance use service need: ^e^				376.51	2	< 0.001
Low likelihood	2021 (34)	1960 (39)	61 (6)			
Moderate likelihood	1901 (32)	1477 (29)	424 (44)			
High likelihood	2100 (35)	1629 (32)	471 (49)			
Current or past month substance use patterns:						
Regular alcohol use: ^f^				138.76	3	< 0.001
Never tried	732 (12)	720 (14)	12 (1)			
Irregular	3963 (66)	3241 (64)	722 (75)			
Regular	462 (8)	364 (7)	98 (10)			
Missing	865 (14)	741 (15)	124 (13)			
Regular cannabis use: ^f^				207.77	3	< 0.001
Never tried	1391 (23)	1331 (26)	60 (6)			
Irregular	1919 (32)	1575 (31)	344 (36)			
Regular	1690 (28)	1303 (26)	387 (40)			
Missing	1022 (17)	857 (17)	165 (17)			
Past month prescription drug use: ^c^				3.60	2	0.165
Yes	560 (9)	467 (9)	93 (10)			
No	4695 (78)	3936 (78)	759 (79)			
Missing	767 (13)	663 (13)	104 (11)			
Past month illicit drug use: ^d^				14.98	2	0.001
Yes	767 (13)	611 (12)	156 (16)			
No	4488 (74)	3792 (75)	696 (73)			
Missing	767 (13)	663 (13)	104 (11)			
GAIN-SS past month likelihood of substance use service need: ^e^				186.99	2	< 0.001
Low likelihood	2992 (50)	2710 (53)	282 (29)			
Moderate likelihood	1807 (30)	1392 (27)	415 (43)			
High likelihood	1223 (20)	964 (19)	259 (27)			

*Table Abbreviations: DF* degrees of freedom, *GAIN-SS* Global Appraisal of Individual Needs- Short Screener

*Table Notes:* (a) Gender diverse/other category includes trans male/female, non-binary, agender, two-spirit, and other; (b) Youth were asked to select all race/ethnicities they identify with. Non-white includes Indigenous, Chinese, Filipino, Japanese, Korean, South Asian, Southeast Asian, West Asian, Latin American, Black, and Arab; (c) Prescription drugs combine any use of painkillers (T3s, Oxy), sedatives (ativan, valium), and/or stimulants (dex, Ritalin); (d) Illicit drugs combine any use of cocaine/crack, amphetamines, heroin/fentanyl, hallucinogens, and/or MDMA; (e) Derived from the substance use subscale of the Global Appraisal of Individual Needs – Short Screener (GAIN-SS). Sub-scale scores range from 0 to 5 for both past month and past year screeners. According to scale norms, 0 symptoms is considered low likelihood of substance use service need, 1–2 symptoms is moderate likelihood of substance use service need, and 3+ symptoms is considered high likelihood of substance use service need; (f) Regular alcohol and cannabis use are collected using the questions: “I would describe my alcohol/cannabis use as regular/daily”

**Table 2:** Risk and protective factors for substance use among youth accessing integrated youth services during the pre- and pandemic-periods

Characteristic	Total Overall	Pre-pandemic	Pandemic	Chi-square	DF	*p-*value
	n = 6022 No. (%)	n = 5066 No. (%)	n = 956 No. (%)			
Social and environmental risk/protective factors						
Education and employment status:				12.62	2	0.002
In education and/or employment	4865 (81)	4098 (81)	767 (80)			
Not in education or employment	877 (14)	715 (14)	162 (17)			
Missing	280 (5)	253 (5)	27 (3)			
Money situation causes stress:				17.06	4	0.002
Never	1202 (20)	1040 (20)	162 (17)			
Sometimes	1850 (31)	1555 (31)	295 (31)			
Often	1148 (19)	939 (18)	209 (22)			
Always	1502 (25)	1247 (25)	255 (27)			
Missing	320 (5)	285 (6)	35 (4)			
I can talk to someone in family if I have problems:				41.81	3	< 0.001
Yes, about most things	1808 (30)	1584 (31)	224 (23)			
Sometimes, depending on the problem	3071 (51)	2540 (50)	531 (55)			
No	894 (15)	715 (14)	179 (19)			
Missing	249 (4)	227 (5)	22 (2)			
Current housing situation: ^a^				11.52	2	0.003
Secure housing	5290 (88)	4421 (87)	869 (91)			
Insecure housing	498 (8)	433 (8)	65 (7)			
Missing	234 (4)	212 (4)	22 (2)			
Feel safe in current living situation:				11.96	2	0.002
Yes	5075 (84)	4234 (83)	841 (88)			
No	554 (9)	484 (10)	70 (7)			
Missing	393 (7)	348 (7)	45 (5)			
Seen or experienced violence in last 3 months:				89.69	2	< 0.001
Yes	2065 (34)	1610 (32)	455 (48)			
No	3632 (60)	3168 (62)	464 (48)			
Missing	325 (5)	288 (6)	37 (4)			
Time per week doing meaningful activities:				13.29	5	0.021
< 2 hours	1015 (17)	868 (17)	147 (15)			
2–5 hours	1417 (23)	1174 (23)	243 (25)			
6–10 hours	1225 (20)	1042 (21)	183 (19)			
11–15 hours	775 (13)	635 (12)	140 (15)			
> 16 hours	1237 (20)	1033 (20)	204 (21)			
Missing	353 (6)	314 (6)	39 (4)			
Health-related risk/protective factors						
Self-rated physical health:				85.85	4	< 0.001
Poor	581 (10)	463 (9)	118 (12)			
Fair	1970 (33)	1614 (32)	356 (37)			
Good	2157 (36)	1802 (36)	355 (37)			
Very good/Excellent	840 (14)	723 (14)	117 (12)			
Missing	474 (8)	464 (9)	10 (1)			
Self-rated mental health:				85.85	4	< 0.001
Poor	2325 (39)	1861 (37)	464 (48)			
Fair	2309 (38)	1924 (38)	385 (40)			
Good	682 (11)	605 (12)	77 (8)			
Very good/Excellent	203 (3)	189 (4)	14 (1)			
Missing	503 (8)	487 (10)	16 (2)			

*Table Abbreviations: DF* degrees of freedom

*Table Notes:* (a) Secure housing includes a house or apartment; Insecure housing includes homeless shelter, street homelessness, single room occupancy hotel, or group home

As shown in Table 2, significant differences were observed in the distribution of the select risk/protective factors between the pre- and pandemic periods. During the pre-pandemic period, a higher proportion of youth were able to talk to family members about their problems (31% vs. 23%) compared to the pandemic period. On the other hand, during the pandemic, a higher proportion of youth reported seeing or experiencing violence (48% vs. 32%) and self-rated their physical (12% vs. 9%) and mental health (48% vs. 37%) as poor compared to the pre-pandemic.

### Effect of COVID-19 pandemic on average number of substance use symptoms

**Fig. 1:**
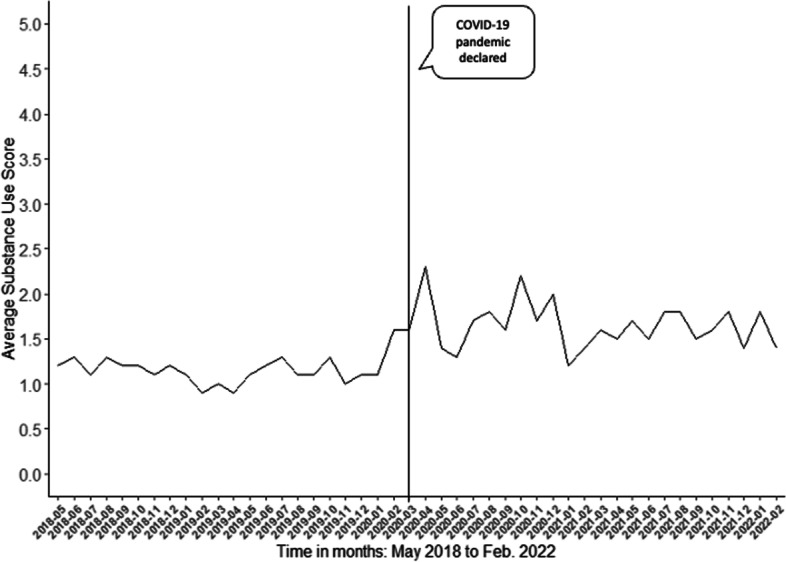
Monthly time series of average past month substance use symptoms, as measured using the GAIN-SS. Scores range from 0 to 5, with higher scores indicating a higher number of substance use symptoms in the past month. Data shown from May 2018 to February 2022, with the line marked at March 2020, when the global COVID-19 pandemic was declared. This line distinguishes the pre- and pandemic periods of the study

Figure 1 shows the monthly time series of average number of past month substance use symptoms endorsed (range 0–5 symptoms) per youth. This figure suggests a sudden increase in the average past month substance use symptoms in the months immediately following the COVID-19 pandemic. Table 3 presents the ITS regression model analyzing whether the pandemic was associated with a change in the average monthly substance use symptoms. These results indicate a positive, but not statistically significant, pre-pandemic trend in substance use symptoms (*p* = 0.384), suggesting no significant change over time during this period. After accounting for the pre-pandemic trend, the level change in the month immediately after the pandemic significantly increased by an average of 0.770 (*p* = 0.01) substance use symptoms per youth. However, there was no significant change in the average monthly trend of substance use symptoms after the pandemic compared to the pre-pandemic period (*p* = 0.242).

**Table 3:** Parameter estimates, standard errors and *p*-values from interrupted time series model predicting average monthly substance use symptoms per youth over time

	Coefficient	Standard error	Z-Score	*p-*value
Intercept	1.102	0.101	10.89	< 0.001
Pre-pandemic trend	0.006	0.007	0.88	0.384
Level change immediately after the pandemic	0.770	0.286	2.69	0.01
Trend change after the pandemic	−0.012	0.010	−1.19	0.242

### Association between risk/protective factors on youths’ alcohol/drug use service need overall and by pandemic period

Table 4 presents results from the multivariable logistic regression analysis for the overall, pre-pandemic and pandemic samples. For the overall sample, the odds of moderate/high likelihood of alcohol/drug use service need were significantly higher during the pandemic compared to pre-pandemic periods, adjusting for age, gender and race (adjusted odds ratio (aOR) = 2.39, 95% confidence interval (95% CI) = 2.04, 2.80). Additionally, the odds of moderate/high likelihood of alcohol/drug use service need were highest for those who saw or experienced violence in the last 3 months (aOR = 2.64; 95% CI = 2.33, 2.99) compared to those who did not. Youth engaging in 6–10 hours (aOR = 0.82; 95% CI = 0.70, 0.98) and 2–5 hours (aOR = 0.84; 95% CI = 0.71, 0.99) of activities had a significantly lower odds of moderate/high likelihood of alcohol/drug use service need compared to those engaging in > 16 hours per week. The adjusted odds of self-rated health on moderate/high likelihood of alcohol/drug use service need were nearly two times higher for youth with poor self-rated physical (aOR = 2.00, 95% CI = 1.54, 2.58) and mental health (aOR = 1.66; 95% CI = 1.18, 2.34). As shown in the stratified multivariable logistic regression results (Table 4), the direction and magnitude of the adjusted associations for these risk/protective factors was consistent in the pre-pandemic-period. However, in the pandemic period, the strength of the adjusted associations for these risk/protective factors decreased, except for poor physical health, which remained statistically significantly associated with a higher adjusted odds of alcohol/drug use service need (aOR = 2.26, 95% CI = 1.14, 4.44).

**Table 4:** Multivariable logistic regression of risk and protective factors associated with moderate/high likelihood of alcohol or drug use service need among youth, stratified by pre-pandemic and pandemic periods

Characteristic	Overall	Pre-pandemic Period	Pandemic Period
	N = 6022	N = 5066	N = 956
	aOR (95% CI)	aOR (95% CI)	aOR (95% CI)
Pandemic vs. Pre-pandemic	**2.39 (2.04, 2.80)**	–	–
Age group			
10–14	1.00	1.00	1.00
15–18	**2.28 (1.87, 2.77)**	**2.86 (2.29, 3.57)**	0.68 (0.39, 1.20)
19–24	**3.85 (3.16, 4.69)**	**5.27 (4.21, 6.60)**	0.75 (0.42, 1.32)
Gender identity			
Woman	1.00	1.00	1.00
Man	**1.39 (1.23, 1.57)**	**1.35 (1.18, 1.54)**	**1.65 (1.18, 2.26)**
Gender diverse/other	**0.71 (0.58, 0.87)**	**0.64 (0.51, 0.80)**	0.97 (0.60, 1.55)
Non-white vs. White race	0.90 (0.80, 1.02)	**0.88 (0.77, 1.00)**	1.31 (0.77, 1.40)
In education and/or employment, no vs. yes	0.91 (0.77, 1.07)	**0.84 (0.70, 1.00)**	1.23 (0.81, 1.87)
I can talk to someone in family if I have problems			
Yes	1.00	1.00	1.00
Sometimes	**1.15 (1.02, 1.31)**	**1.21 (1.05, 1.40)**	0.95 (0.69, 1.36)
No	1.07 (0.90, 1.29)	1.05 (0.86, 1.28)	1.10 (0.69, 1.75)
Feel safe in current living situation, no vs. yes	1.00 (0.82, 1.23)	0.98 (0.79, 1.22)	1.17 (0.61, 2.21)
Seen or experienced violence in last 3 months, yes vs. no	**2.64 (2.33, 2.99)**	**3.06 (2.67, 3.52)**	1.28 (0.94, 1.74)
Time per week doing meaningful activities			
> 16 hours	1.00	1.00	1.00
11–15 hours	0.97 (0.80, 1.18)	0.98 (0.79, 1.21)	1.30 (0.64, 1.67)
6–10 hours	**0.82 (0.70, 0.98)**	**0.78 (0.65, 0.94)**	1.11 (0.70, 1.74)
2–5 hours	**0.84 (0.71, 0.99)**	**0.83 (0.69, 1.00)**	0.95 (0.62, 1.45)
< 2 hours	0.86 (0.72, 1.04)	0.86 (0.81, 1.05)	0.96 (0.58, 1.59)
Self-rated physical health			
Excellent/Very good	1.00	1.00	1.00
Good	**1.27 (1.06, 1.52)**	**1.28 (1.05, 1.56)**	1.20 (0.75, 1.92)
Fair	**1.63 (1.35, 1.98)**	**1.74 (1.41, 2.15)**	1.15 (0.71, 1.86)
Poor	**2.00 (1.54, 2.58)**	**1.95 (1.47, 2.58)**	**2.26 (1.15, 4.44)**
Self-rated mental health			
Excellent/Very good	1.00	1.00	1.00
Good	0.99 (0.70, 1.41)	0.93 (0.64, 1.36)	1.50 (0.45, 5.00)
Fair	1.33 (0.95, 1.86)	1.29 (0.90, 1.84)	1.74 (0.56, 5.43)
Poor	**1.66 (1.18, 2.34)**	**1.57 (1.09, 2.25)**	2.39 (0.76, 7.57)

*Table Notes: aOR* adjusted odds ratio; 95% CI = 95% confidence interval; bolded text denotes statistically significant effects where *p* < 0.05

## Discussion

In this repeated monthly cross-sectional study of youth accessing a network of IYS, we observed a significant increase in youths’ alcohol or drug use immediately after the COVID-19 pandemic was declared in March 2020, with substance use returning to pre-pandemic trends in the months thereafter. Despite significant changes in youths’ risk/protective factors for substance use during the pandemic, their effects remained relatively consistent across the pre- and pandemic-periods. These findings contribute original evidence regarding the full effects of the pandemic on youth substance use and its risk/protective factors and inform future research, interventions, and policy.

To date, research regarding the impacts of the pandemic on youth substance use has been quite inconsistent, including among studies that have used designs and combined measures of alcohol or drug use (e.g., cannabis, illicit drugs, non-prescribed use of prescription drugs, etc) similar to our study [9]. For instance, one cross-sectional study measuring alcohol, cannabis, or drug use, reported an increase in alcohol and non-specified drug use during the pandemic [23], while another reported less alcohol, cannabis and other drug use [24]. To our knowledge, two other studies [25, 26] have used the same validated outcome measure (i.e., GAIN-SS for past 30 days) as in our study. The first of these was conducted approximately 3 weeks after the pandemic was declared and reported a decrease in substance use behaviours [25]. The second study examined trajectories of substance use from April to October 2020 and reported relatively stable substance use behaviours across groups with low, moderate, or high levels of alcohol or drug use service need [26]. In contrast, our study indicates that the pandemic had an immediate effect on substance use behaviours and was associated with a two-fold increase in moderate/high likelihood of alcohol or drug use service need. These discrepancies may be due to differences in the study’s observation periods and related exposure definitions (e.g., early in pandemic, later in pandemic, etc). Therefore, a key strength of our study was the use of monthly time series data. This allowed comparison of monthly changes in substance use for the 22-month pre-pandemic and 24-month pandemic periods; thereby providing a longer and more balanced observation period to examine these impacts.

Our study also provides further insight into the risk and protective factors that were associated with the greatest need for alcohol or drug use services. For the overall study sample, our study determined significant independent associations for risk/protective factors, including being able to talk to a family member about problems, seeing or witnessing violence, engagement in meaningful activities, and self-rated physical and mental health. These findings are consistent with longstanding evidence regarding the crucial role that such individual and interpersonal factors have on youths’ substance use patterns [5]. For example, it is widely recognized that family connection is a protective factor, with prior research showing family support can reduce the risk of substance use disorders by approximately 50% [27]. Conversely, adverse childhood experiences, such as parental emotional or physical abuse, are known risk factors for substance use among youth and adult populations [5, 28, 29]. In our study, being able to talk to a family member sometimes and seeing or witnessing violence were two conceptually related interpersonal factors that were not collinear. This suggests that such interpersonal factors can have differential effects on the likelihood of substance use service need (i.e., the presence of one experience does not guarantee the absence of another) and demonstrates the complexity of their effects on youth substance use and the benefit of conducting research into their causal pathways.

Accordingly, another important contribution of our research was the stratified regression analyses, which can inform the potential moderating role of the pandemic on the association between the risk and protective factors and likelihood of service need. Our study’s stratified regression analyses determined that the associations of these risk and protective factors and service need were similar in direction between the pre- and pandemic-periods, suggesting homogeneity of effects. Interestingly, the strength of the associations between the risk and protective factors and service need diminished in the pandemic period, possibly due to its lower statistical power. For instance, exposure to violence was positively, though not significantly, associated with alcohol or drug use service need in the pandemic period. While these findings should be considered exploratory, our overall findings and the stratified analyses complement those few studies that have similarly considered risk and protective factors and substance use during the pandemic [14–16].

Building on these studies, our study also identified significant independent associations between self-rated physical health and meaningful activities with substance use service need. Of note, youth engaging in less hours per week of meaningful activities (2–10 hours vs. > 16 hours) had lower odds of moderate/high substance use service need. This unexpected finding held when we checked hours per week of physical exercise, a closely related variable. It is possible that youth using substances were engaging in meaningful activities (e.g., physical exercise) to reduce their substance use, an explanation that is supported by some research among adults accessing treatment for substance use [30, 31]. Though further research is needed, another explanation is that youth were using substances while engaging in meaningful activities, such as sports and clubs, given evidence that social contexts for substance use changed during the pandemic [16]. As our cross-sectional data are unable to tease such associations apart, future research should consider repeated measures designs that allow for within-person analyses (e.g., growth curve models) to disentangle the complex relationship between time-varying individual and interpersonal risk/protective factors and substance use service need.

There are considerable health services and policy implications of our research. At a service delivery-level, the strong and consistent associations between the risk/protective factors and alcohol or drug use service need provide compelling evidence to routinely assess these factors over time, particularly as they are sensitive to change. Thus, a direct recommendation from our study is that clinicians working with youth consider how risk/protective factor frameworks [6, 18] could be systematically integrated into routine visits to identify youth that may benefit from further screening, assessment, harm reduction and/or treatment for substance use. While numerous practice and policy-related barriers would need to be addressed to implement such practices (e.g., clinician training, resources), this would allow earlier identification of substance use [32].

For policy makers, it is also critical to note that 50% of youth met moderate/high likelihood of past month substance use service need, with the prevalence being significantly impacted by the pandemic. This signals an urgent need for increased population-level approaches, standards, and infrastructure. Indeed, IYS provide one such opportunity and are increasingly recognized as a key innovation towards reducing gaps in youth mental health care. However, substance use is one of five service streams offered within IYS and additional resources are needed to increase the number of trained professionals and the scope of evidence-based substance use services in these settings. Additionally, where IYS are not yet available, increased capacity in existing youth services, including peer support, and expansion of youth-specific substance use services, will be critical to meet current levels of need.

### Strengths and limitations

As noted, there are several strengths to our study, including the large PROs data set, use of a validated clinical outcome measure, and four-year observation period. However, there are some important limitations to be considered. First, our data were drawn from a non-random sample of youth who sought or received IYS and voluntarily completed PROs close to the time of their first IYS visit. As a result, the data are not likely representative of the general youth population and data on important confounders (e.g., geographic location, socio-economic status) were not available for this study. Thus, our hypotheses were exploratory and not registered a priori. This limits our ability to draw population-level inferences about the study’s findings and the full scope of policy implications (e.g., primary prevention programs). Additionally, as the pandemic was accompanied by changes in service delivery (e.g., fewer in-person visits), this led to lower rates of PROs completion during the initial months of the pandemic and the unbalanced pre- and pandemic sample sizes. Despite these changes, a sensitivity analysis (Additional file 1, Table 4) confirmed few significant differences between PROs completed early (March – May 2020) and later in the pandemic (March – May 2021). Nevertheless, the slightly higher item non-response during the pandemic period may have resulted in unmeasured confounding that was only partially adjusted for with the missing indicator method [22].

## Conclusion

Our research indicates that the COVID-19 pandemic was associated with a significant increase in alcohol or drug use and had an immediate impact on alcohol or drug use service need among youth accessing integrated youth services. Although the study observed changes to known risk and protective factors for substance use, the pandemic did not appear to modify their effect on substance use, suggesting these factors remain crucial to prevention, early identification and intervention efforts. These findings respond to critical empirical gaps regarding the impact of the COVID-19 pandemic on youth substance use and inform directions for research, health services and policy.

## Supplementary Information

**Additional file 1.**

## Data Availability

Data for this study are not publicly available as participants of this study did not agree for their data to be shared publicly.

## Abbreviations

COVID-19: coronavirus disease
IYS: integrated youth services
BC: British Columbia
PROs: patient reported outcomes
GAIN-SS: Global Appraisal of Individual Needs – Short Screener
ITS: interrupted time series
