# Supplementary material for: Impact of the COVID-19 pandemic on alcohol or drug use symptoms and service need among youth: a cross-sectional sample from British Columbia, Canada
Source: Subst Abuse Treat Prev Policy. 2022 Dec 22;17:82. doi: 10.1186/s13011-022-00508-9 (PMC9774070; doi:10.1186/s13011-022-00508-9)
Supplement: Supplementary file 1 — Additional file 1. [file 13011_2022_508_MOESM1_ESM.docx]

**Supplementary Material**

**Table 1. Univariable logistic regression of risk and protective factors associated with moderate/high likelihood of alcohol or drug use service need among youth (n=6,022)**

| **Risk and Protective Factors** | **OR** | **95% CI** | | **p-value** | |
| --- | --- | --- | --- | --- | --- |
|  |  | **Lower limit** | **Upper limit** | |  |
| Pandemic vs. Pre-pandemic | 2.75 | 2.37 | 3.19 | | <0.001 |
| Age group: |  |  |  | |  |
| 10-14 | 1.00 | - | - | | - |
| 15-18 | 2.20 | 1.83 | 2.63 | | <0.001 |
| 19-24 | 3.39 | 2.84 | 4.05 | | <0.001 |
| Gender identity: |  |  |  | |  |
| Woman | 1.00 | - | - | | - |
| Man | 1.45 | 1.30 | 1.62 | | <0.001 |
| Gender diverse/other | 0.88 | 0.74 | 1.06 | | 0.18 |
| Non-white vs. White race | 0.96 | 0.86 | 1.07 | | 0.46 |
| In education and/or employment, no vs.yes | 1.49 | 1.29 | 1.73 | | <0.001 |
| Does your money situation stress you out? |  |  |  | |  |
| Never | 1.00 | - | - | | - |
| Sometimes | 1.76 | 1.51 | 2.04 | | <0.001 |
| Often | 2.52 | 2.14 | 2.98 | | <0.001 |
| Always | 3.72 | 3.17 | 4.37 | | <0.001 |
| I can talk to someone in family if I have problems: |  |  |  | |  |
| Yes | 1.00 | - | - | | - |
| Sometimes | 1.26 | 1.12 | 1.41 | | 0.00 |
| No | 1.36 | 1.16 | 1.60 | | 0.00 |
| Current housing type: |  |  |  | |  |
| Secure | 1.00 | **-** | **-** | | **-** |
| Insecure | 1.19 | 0.99 | 1.43 | | 0.07 |
| Feel safe in current living situation, no vs. yes | 1.34 | 1.12 | 1.60 | | 0.00 |
| Seen or experienced violence in last three months, yes vs. no | 2.64 | 2.36 | 2.95 | | <0.001 |
| Time per week doing meaningful activities: |  |  |  | |  |
| >16 hours | 1.00 | - | - | | - |
| 11-15 hours | 0.94 | 0.78 | 1.12 | | 0.48 |
| 6-10 hours | 0.80 | 0.68 | 0.93 | | 0.00 |
| 2-5 hours | 0.86 | 0.74 | 1.00 | | 0.06 |
| <2 hours | 1.01 | 0.85 | 1.19 | | 0.93 |
| Self-rated physical health: |  |  |  | |  |
| Excellent/Very good | 1.00 | - | - | | - |
| Good | 1.43 | 1.22 | 1.68 | | <0.001 |
| Fair | 2.12 | 1.80 | 2.50 | | <0.001 |
| Poor | 3.21 | 2.57 | 4.00 | | <0.001 |
| Self-rated mental health: |  |  |  | |  |
| Excellent/Very good | 1.00 | - | - | | - |
| Good | 1.07 | 0.77 | 1.48 | | 0.68 |
| Fair | 1.70 | 1.26 | 2.29 | | 0.00 |
| Poor | 2.46 | 1.83 | 3.32 | | <0.001 |

**Table Notes:** OR= adjusted odds ratio; 95% CI = 95% confidence interval

**Table 2. Contrasts test of overall effect of independent variables with > 2 categories in the stratified multivariable logistic regression analysis**

|  | **Overall**  **N=6022** | | | **Pre-pandemic Period N=5066** | | | **Pandemic Period N=956** | | |
| --- | --- | --- | --- | --- | --- | --- | --- | --- | --- |
| **Contrast** | **DF** | **Chi-Square^a^** | **p-value** | **DF** | **Chi-Square^a^** | **p-value** | **DF** | **Chi-Square^a^** | **p-value** |
| **Age group** |  |  |  |  |  |  |  |  |  |
| (10-14 omitted) |  |  |  |  |  |  |  |  |  |
| 15-18 | 1 | 178.87 | <0.001 | 1 | 178.87 | <0.001 | 1 | 1.75 | 0.19 |
| 19-24 | 1 | 15.33 | <0.001 | 1 | 15.33 | <0.001 | 1 | 1.00 | 0.32 |
| **Gender identity** |  |  |  |  |  |  |  |  |  |
| (Woman omitted) |  |  |  |  |  |  |  |  |  |
| Man | 1 | 28.50 | <0.001 | 1 | 28.50 | <0.001 | 1 | 8.83 | 0.00 |
| Gender diverse/other | 1 | 11.19 | 0.00 | 1 | 11.19 | 0.00 | 1 | 0.02 | 0.89 |
| **I can talk to someone in family if I have problems** | | | | | | | | | |
| (Yes, about most things' omitted) |  |  |  |  |  |  |  |  |  |
| Sometimes, depending on the problems | 1 | 4.77 | 0.03 | 1 | 4.77 | 0.03 | 1 | 0.07 | 0.79 |
| No | 1 | 0.59 | 0.44 | 1 | 0.59 | 0.44 | 1 | 0.16 | 0.69 |
| **Time per per week doing meaningful activities** | | | | | | | | | |
| ('16 hours +' omitted) |  |  |  |  |  |  |  |  |  |
| 11-15 hours | 1 | 0.08 | 0.78 | 1 | 0.08 | 0.78 | 1 | 0.02 | 0.89 |
| 6-10 hours | 1 | 4.79 | 0.03 | 1 | 4.79 | 0.03 | 1 | 0.19 | 0.66 |
| 2-5 hours | 1 | 4.24 | 0.04 | 1 | 4.24 | 0.04 | 1 | 0.06 | 0.81 |
| Less than 2 hours | 1 | 2.51 | 0.11 | 1 | 2.51 | 0.11 | 1 | 0.02 | 0.89 |
| **Self-rated physical health** |  |  |  |  |  |  |  |  |  |
| ('Excellent/very good' omitted) |  |  |  |  |  |  |  |  |  |
| Good | 1 | 6.57 | 0.01 | 1 | 6.57 | 0.01 | 1 | 0.60 | 0.44 |
| Fair | 1 | 24.71 | <0.001 | 1 | 24.71 | <0.001 | 1 | 0.32 | 0.57 |
| Poor | 1 | 27.89 | <0.001 | 1 | 27.89 | <0.001 | 1 | 5.61 | 0.02 |
| **Self-rated mental health** |  |  |  |  |  |  |  |  |  |
| ('Excellent/very good' omitted) |  |  |  |  |  |  |  |  |  |
| Good | 1 | 0.00 | 0.96 | 1 | 0.00 | 0.96 | 1 | 0.44 | 0.51 |
| Fair | 1 | 2.79 | 0.09 | 1 | 2.79 | 0.09 | 1 | 0.91 | 0.34 |
| Poor | 1 | 8.49 | 0.00 | 1 | 8.49 | 0.00 | 1 | 2.21 | 0.14 |

**Table Notes:** DF= degrees of freedom; (a) the Wald Chi-Square indicates the strength of the association between one category (e.g., poor mental health Chi-square = 8.49, df=1, p-value = 0.004) with the other categories (e.g., good and fair mental health), regardless of the reference category being in the model. The p-value indicates if it is a statistically significant association.

**Table 3. Complete case analysis of multivariable logistic regression of risk and protective factors associated with moderate/high likelihood of substance use service need among youth, stratified by pre-pandemic and pandemic periods**

| **Characteristic** | **Overall** | **Pre-pandemic Period** | **Pandemic Period** |
| --- | --- | --- | --- |
|  | **N= 4911** | **N= 4074** | **N= 837** |
|  | **aOR (95% CI)** | **aOR (95% CI)** | **aOR (95% CI)** |
| Pandemic vs. Pre-pandemic | **2.32 (1.96, 2.76)** |  |  |
| Age group: |  |  |  |
| 10-14 | 1.00 | 1.00 | 1.00 |
| 15-18 | **2.46 (1.98, 3.07)** | **3.14 (2.44, 4.04)** | 0.59 (0.30, 1.13) |
| 19-24 | **4.17 (3.34, 5.21)** | **5.77 (4.48, 7.43)** | 0.67 (0.34, 1.30) |
| Gender identity: |  |  |  |
| Woman | 1.00 | 1.00 | 1.00 |
| Man | **1.38 (1.21, 1.58)** | **1.33 (1.15, 1.54)** | **1.61 (1.14, 2.27)** |
| Gender diverse/other | **0.73 (0.59, 0.91)** | **0.64 (0.49, 0.82)** | 1.06 (0.63, 1.76) |
| Non-white vs. White race | 0.89 (0.78, 1.02) | **0.85 (0.74, 0.98)** | 1.13 (0.82, 1.55) |
| In education and/or employment, no vs.yes | 0.86 (0.72, 1.03) | **0.79 (0.65, 0.96)** | 1.11 (0.72, 1.72) |
| I can talk to someone in family if I have problems: |  |  |  |
| Yes | 1.00 | 1.00 | 1.00 |
| Sometimes | **1.18 (1.03, 1.36)** | **1.25 (1.07, 1.45)** | 0.91 (0.63, 1.33) |
| No | 1.12 (0.91, 1.36) | 1.13 (0.91, 1.40) | 0.96 (0.59, 1.57) |
| Feel safe in current living situation, no vs. yes | 1.05 (0.84, 1.31) | 1.05 (0.83, 1.33) | 1.10 (0.57, 2.11) |
| Seen or experienced violence in last three months, yes vs. no | **2.65 (2.31, 3.03)** | **3.10 (2.66, 3.60)** | 1.27 (0.92, 1.75) |
| Time per week doing meaningful activities: |  |  |  |
| >16 hours | 1.00 | 1.00 | 1.00 |
| 11-15 hours | 0.97 (0.78, 1.19) | **0.80 (0.65, 0.97)** | 1.09 (0.66, 1.80) |
| 6-10 hours | **0.80 (0.67, 0.97)** | **0.74 (0.61, 0.91)** | 1.06 (0.66, 1.70) |
| 2-5 hours | **0.82 (0.68, 0.98)** | 0.95 (0.76, 1.20) | 0.95 (0.61, 1.48) |
| <2 hours | **0.81 (0.67, 0.99)** | **0.79 (0.63, 0.98)** | 0.99 (0.59, 1.69) |
| Self-rated physical health: |  |  |  |
| Excellent/Very good | 1.00 | 1.00 | 1.00 |
| Good | **1.24 (1.03, 1.51)** | **1.27 (1.03, 1.58)** | 1.04 (0.63, 1.71) |
| Fair | **1.58 (1.28, 1.94)** | **1.72 (1.37, 2.16)** | 0.96 (0.57, 1.60) |
| Poor | **1.85 (1.41, 2.43)** | **1.81 (1.34, 2.44)** | 1.92 (0.94, 3.92) |
| Self-rated mental health: |  |  |  |
| Excellent/Very good | 1.00 | 1.00 | 1.00 |
| Good | 1.10 (0.75, 1.59) | 1.02 (0.68, 1.52) | 1.59 (0.45, 5.62) |
| Fair | 1.37 (0.96, 1.96) | 1.35 (0.93,1.97) | 1.45 (0.44, 4.75) |
| Poor | **1.74 (1.21, 2.50)** | **1.64 (1.12, 2.41)** | 2.19 (0.66, 7.28) |

**Table Notes:** aOR= adjusted odds ratio; 95% CI = 95% confidence interval; bolded text denotes statistically significant effects where *p*-value <0.05

**Table 4. Comparison of sample characteristics early (March – May 2020) and later (March – May 2021) in the pandemic**

|  | **Pandemic Samples** | | |
| --- | --- | --- | --- |
|  | **Early in the pandemic ^a^**  **(N=97)** | **Later in the pandemic ^b^ (N=121)** | **Total**  **(N=218)** |
| **Past month likelihood of substance use service need:** | N (%) | N (%) | N (%) |
| Low likelihood | 28 (29) | 41 (34) | 69 (32) |
| Moderate/high likelihood | 69 (71) | 80 (66) | 149 (68) |
| **Age group: ***** |  |  |  |
| 10-14 | NA (1) | 14 (12) | 15 (7) |
| 15-18 | 26 (27) | 54 (45) | 80 (37) |
| 19-24 | 65 (67) | 53 (44) | 118 (54) |
| Missing | 5 (5) | 0 (0) | 5 (2) |
| **Gender identity:** |  |  |  |
| Woman | 56 (58) | 67 (55) | 123 (56) |
| Man | 31 (32) | 45 (37) | 76 (35) |
| Diverse/other | 10 (10) | 9 (7) | 19 (9) |
| **Race:** |  |  |  |
| White | 65 (67) | 63 (52) | 128 (59) |
| Non-White | 30 (31) | 54 (45) | 84 (38) |
| Missing | NA (2) | NA (3) | 6 (3) |
| **In education and/or employment:** |  |  |  |
| In education and/or employment | 75 (77) | 105 (87) | 180 (83) |
| Not in education or employment | 18 (19) | 15 (12) | 33 (15) |
| Missing | NA (4) | NA (1) | 5 (2) |
| **I can talk to someone in my family if I have problem:** |  |  |  |
| Yes, about most things | 22 (23) | 28 (23) | 50 (23) |
| Sometimes, depending on the problem | 55 (57) | 71 (59) | 126 (58) |
| No | 15 (15) | 22 (18) | 37 (17) |
| Missing | 5 (5) | 0 (0) | 5 (2) |
| **Safe in current living situation:** |  |  |  |
| Yes | 86 (89) | 115 (95) | 201 (92) |
| No | 5 (5) | NA (3) | 9 (4) |
| Missing | 6 (6) | NA (2) | 8 (4) |
| **Seen or experienced violence in last 3 months: *** |  |  |  |
| Yes | 56 (58) | 58 (48) | 114 (52) |
| No | 35 (36) | 61 (50) | 96 (44) |
| Missing | 6 (6) | NA (2) | 8 (4) |
| **Time per week doing meaningful activities:** |  |  |  |
| < 2 hours | 17 (17) | 15 (12) | 32 (15) |
| 2-5 hours | 23 (24) | 35 (29) | 58 (27) |
| 6-10 hours | 19 (20) | 21 (17) | 40 (18) |
| - 1. ours | 9 (9) | 25 (21) | 34 (16) |
| >16 hours | 22 (23) | 23 (19) | 45 (21) |
| Missing | 7 (7) | NA (2) | 9 (4) |
| **Self-rated physical health:** |  |  |  |
| Excellent/very good | 12 (12) | 18 (15) | 30 (14) |
| Good | 30 (31) | 45 (37) | 75 (34) |
| Fair | 42 (43) | 44 (36) | 86 (39) |
| Poor | 13 (13) | 14 (12) | 27 (12) |
| **Self-rated mental health: *** |  |  |  |
| Excellent/very good | 0 (0) | 6 (5) | 6 (3) |
| Good | 11 (11) | 6 (5) | 17 (8) |
| Fair | 32 (33) | 56 (46) | 88 (40) |
| Poor | 52 (54) | 52 (43) | 104 (48) |
| Missing | NA (2) | NA (1) | NA (1) |

**Table Notes:** **p*<0.05, ***p*<0.01; ****p*<0.001; *p*-values derived from chi-square tests; (a) Youth completing patient reported outcomes data between March – May 2020; (b) Youth completing patient reported outcomes data between March – May 2021.
